# The Design, Fabrication, and Testing of an Electromagnetic Micropump with a Matrix-Patterned Magnetic Polymer Composite Actuator Membrane

**DOI:** 10.3390/mi9010013

**Published:** 2017-12-31

**Authors:** Muzalifah Mohd Said, Jumril Yunas, Badariah Bais, Azrul Azlan Hamzah, Burhanuddin Yeop Majlis

**Affiliations:** 1 Institute of Microengineering and Nanoelectronics (IMEN), Universiti Kebangsaan Malaysia (UKM), 43600 UKM Bangi, Selangor, Malaysia; muzalifah@utem.edu.my (M.M.S.); azlanhamzah@ukm.edu.my (A.A.H.); burhan@ukm.edu.my (B.Y.M.); 2 Faculty of Electronics and Computer Engineering (FKEKK), Universiti Teknikal Malaysia Melaka (UTeM), Hang Tuah Jaya, 76100 Durian Tunggal, Melaka, Malaysia; 3 Dept. of Electrical Electronic and Systems Engineering, Faculty of Engineering and Built Environment, 43600 UKM Bangi, Selangor, Malaysia; badariah@ukm.edu.my

**Keywords:** valveless electromagnetic (EM) micropump, polydemethylsiloxane (PDMS), NdFeB, polymer composite membrane, magnetic actuator, drug delivery

## Abstract

A valveless electromagnetic (EM) micropump with a matrix-patterned magnetic polymer composite actuator membrane structure was successfully designed and fabricated. The composite membrane structure is made of polydemethylsiloxane (PDMS) that is mixed with magnetic particles and patterned in matrix blocks. The matrix magnetic composite membrane was fabricated using a soft lithography process and expected to have a compact structure having sufficient magnetic force for membrane deformation and maintained membrane flexibility. The magnetic membrane was integrated with the microfluidic system and functionally tested. The experimental results show that a magnetic composite actuator membrane containing of 6% NdFeB is capable of producing a maximum membrane deflection up to 12.87 µm. The functionality test of the EM actuator for fluid pumping resulted in an extremely low sample injection flow rate of approximately 6.523 nL/min. It was also concluded that there is a correlation between the matrix structure of the actuator membrane and the fluid pumping flow rate. The injection flow rate of the EM micropump can be controlled by adjusting the input power supplied to the EM coil, and this is believed to improve the injection accuracy of the drug dosage and have potential in improving the proficiency of the existing drug delivery system.

## 1. Introduction

An electromagnetic (EM) actuator membrane is the most essential mechanical structure in an electromagnetically driven mechanical micropump system since a stable and reliable membrane structure against magnetic force is needed. The EM actuator membrane should be made from soft and elastic material that is able to respond to an electromagnetic field [1,2,3]. Having a reliable and high precision of dosage is needed when the controllable fluidic dosage and flow rate is crucial [1,2,4] such as when a fluid sample is delivered from sample storage into the human body, such as a drug delivery system [2], or into a biological analysis system, such as a lab-on-chip system [5]. Great interest in improving the quality of the mechanical component of the membrane has led to the creation of membrane material that is magnetically responsive, patternable, and highly flexible.

There are many actuation mechanisms that operate the micropump system reported since several years ago, such as electrostatic, piezoelelectric, and thermal pneumatic as well [6]. Among all these reported mechanisms, a micropump driven by electromagnetic actuator seems to have better actuation control and an easy fabrication method. In earlier designs, an EM actuator membrane made of silicon or metal was bonded with a bulky permanent magnet glued to the top of the membrane [7,8,9,10,11]. Those materials are brittle, stiff, and inflexible, especially in a condition of bonding between the thin actuator membrane and a bulky magnet, which can be easily damaged or released during operation.

Researchers have replaced the silicon membrane with polymeric material to generate consistent actuation, as a polymer membrane is physically soft and highly flexible [12,13,14]. However, changing the membrane material to a polymer cannot bear the weight of a bulk permanent magnet. In order to overcome this problem, researchers started to minimize the bulk magnet by manipulating permanent magnetic materials and structure [15,16]. An electroplated CoNiMnP array on a silicon membrane surface was extensively studied in order to find a membrane structure with a high response to the applied magnetic field [16]. Researchers have since started focusing on alternative membrane material. A stiff and brittle membranes have been replaced with a soft and flexible material.

Further study on the flexible membrane has led to a new functional structure and material, achieved by creating an electroplated magnetic layer on a polymer membrane. Research done by Chang et al. [15] minimized the size of the permanent magnet by having an electroplated CoNiMnP layer on the surface of the polydemethylsiloxane (PDMS) diaphragm. Some improvements have been made by Su and Chen; they patterned the electroplated permanent magnet into several smaller pieces of a 7 × 7 matrix of CoNiMnP (50 × 50 × 20 μm^3^) on a square-shaped PDMS membrane. The purpose of the work was to produce a large displacement that will permit a higher volume liquid flow of the micropump [17].

In this work, we replaced the conventional magnetic membrane structure with a new functional material made of PDMS (polydimethylsiloxane) with embedded magnetic particles, which led to a more compact membrane structure with a high magnetic response and improved mechanical deformation capability. PDMS polymers were used due to their good elasticity, high flexibility, high surface strength, and their biocompatibility. Additionally, PDMS can be patterned using a soft lithography process technique [18,19,20].

In this paper, we present a valveless electromagnetic micropump composed with a PDMS-based magnetic-composite polymer actuator membrane with a matrix pattern. Moreover, this paper also reports the operational performance of the fabricated EM actuator and the functionality test of the EM micropump system for fluid flow control in a lab-on-chip and drug delivery system.

## 2. The Design and Fabrication of Electromagnetic (EM) Electromagnetic Actuator

**Figure 1:**
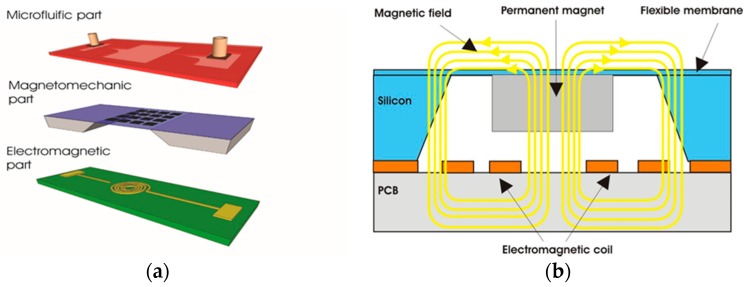
Schematic pictures of the designed valveless electromagnetic (EM) micropump system components: (**a**) the structure of the micropump system; (**b**) the mechanism of electromagnetic actuation.

The EM micropump consists of three main components: a microfluidic component, a magnetomechanic actuator, and an electromagnetic component, as shown in Figure 1a. Studies on the microfluidic and electromagnetic components have been reported elsewhere [2,18,21,22,23]. Therefore, the design of the microcoil, the microfluidic channel, and the chamber will not be discussed in this paper. We focus on the improvement of the magnetomechanic actuator that consists of a permanent magnet and a deformable mechanical membrane that is responsive to the magnetic field generated by the electromagnetic coil. Theoretically, when a magnetic field generated by a microcoil interacts with the magnet particles embedded in the membrane composite, it produces force that causes the composite membrane to deform periodically (Figure 1b) [1,15].

The push–pull action of the membrane in the pump chamber will create a pressure change in the chamber and allow for the transport of the fluids from the inlet to the chamber. The direction of the fluidic flow is determined by the diffuser and nozzle design [14,24,25].

**Figure 2:**
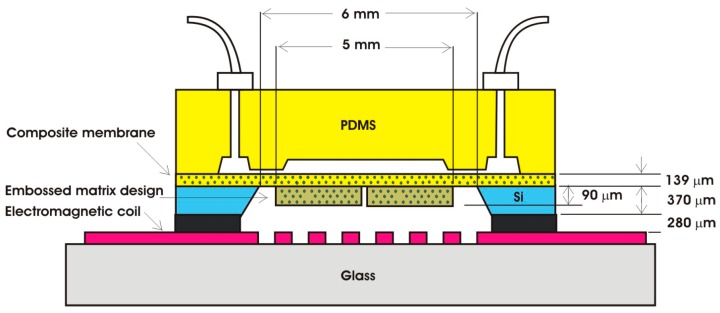
The design of the proposed EM micropump.

Figure 2 shows the design specification of the proposed EM micropump consisting of a matrix-patterned mold structure attached to a silicon substrate and bonded with the PDMS-based microfluidic component. The membrane and the microfluidic component are integrated with the electromagnetic coil to complete the micropump system.

Table 1 shows the specific embossed matrix design of the membrane. Two matrix structure types, namely, square flat and square shapes with dimensions of (1 × 1), (2 × 2), or (3 × 3), were designed to observe the effects of the matrix combination on the deformation capability of the membrane.

### 2.1. Synthesis of Magnetic Polymer Composite Membrane

The polymer was made with a SYLGARD^®^ 184 silicon elastomer kit (Dow Corning, Midland, MI, USA) containing PDMS and curing agent, mixed at a 10:1 ratio. The PDMS mixture was then mixed with 6% NdFeB magnetic particles using a mechanical stirrer.

After the mixing process, the composite magnetic polymer was spin-coated onto a pre-developed SU8 matrix mold structure. The spin coater machine was set at 500 rpm for 10 s to spread the composite. The composite was then ramped up to a higher speed of 800 rpm for 20 s to obtain a membrane thickness of ~139 µm. A thinner membrane layer could be obtained by increasing the coating speed. The spin-coated composite material was finally hard-cured at 60 °C for 30 min to completely remove the remaining water content.

### 2.2. Magnetic Properties of Magnetic Polymer Composite

**Figure 3:**
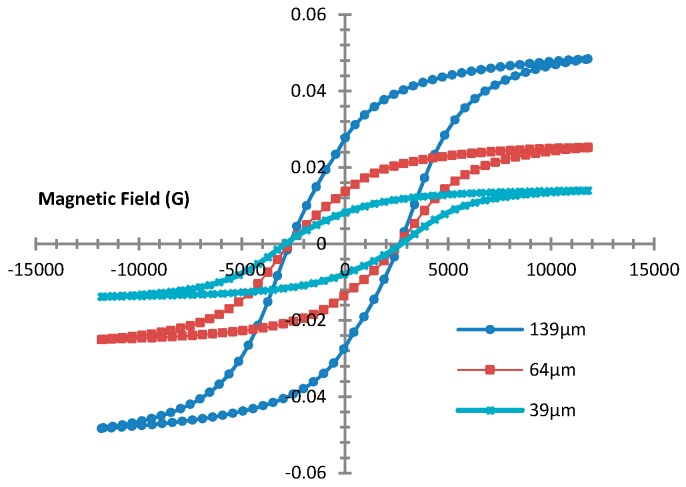
The hysteresis loop of the 6% NdFeB polymer composite.

The magnetic property of the polymer composite was studied by analyzing the hysteresis curve using a vibration sample magnetometer (VSM). Figure 3 shows the hysteresis loop of synthesized PDMS:NdFeB at room temperature and with a magnetization of 1.2 T and a 6% mixture composition, found to be the optimum composition [26]. It can be seen that the magnetic property still exists in the magnetic composite material at different thicknesses.

It can be seen that the layer thickness has a significant effect on the magnetic properties of the composite material. This is probably due to the amount of magnetic particles contained in the polymer matrix. The thicker the layer, the greater the magnetization can be. A 139 µm membrane thickness shows a saturated remanence magnet of 37.637 mT. The coercivity value of the polymer composite is about 2720 G, which is the force required to produce a zero magnetism in a material.

### 2.3. Electro-Mechanical Properties of Magnetic Composite Membrane

**Figure 4:**
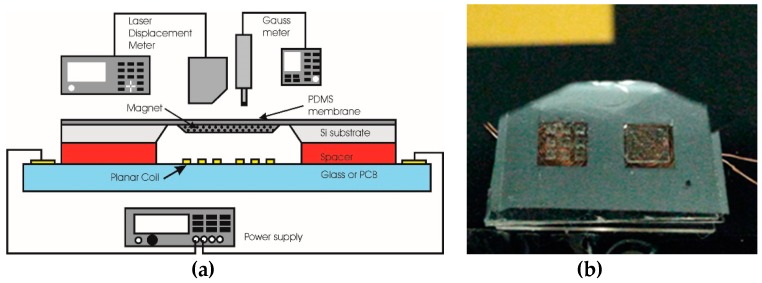
Schematic setup for the measurement of actuator membrane displacement (**a**) and the photograph of the fabricated actuator under test (**b**).

To evaluate the electromechanical properties of the magnetic composite membrane, a membrane deflection test using a laser displacement meter was conducted in open air (without the attachment of the microfluidic component). The measurement system consists of a probing stage, a power supply, a Gaussian meter, and a laser displacement meter. Figure 4 shows the schematic diagram of the membrane deflection measurement setup (a) and the fabricated membrane under the laser probe during the measurement (b).

**Figure 5:**
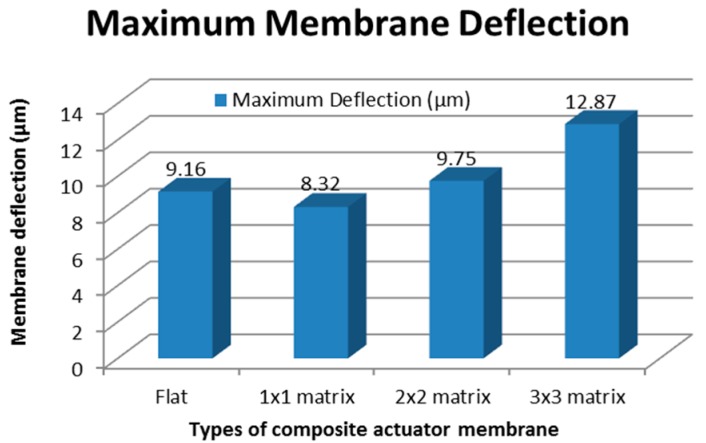
The deflection of the EM actuator membrane when an alternating current (AC) input voltage signal is applied.

For the test, an alternating current (AC) voltage was applied to the electromagnetic for about 60 s until the membrane gave a steady state response. The composite membrane with a varied embossed matrix structure was then measured. The maximum deflection for all membrane types is plotted in Figure 5.

Figure 5 clearly shows that the membrane with a 3 × 3 matrix embossed design contributes to the highest deflection of approximately 12.87 µm. The membrane with an embossed 1 × 1 matrix had the lowest deflection value. This is because the membranes with larger embossed matrices are more rigid, which makes them hard to bend. Smaller cubes make the membrane more flexible, and spaces between matrix cubes are utilized during deformation.

These results confirm an early simulation study done where a membrane with a smaller magnet will produce a higher deflection [27].

## 3. Pump System Integration

**Figure 6:**
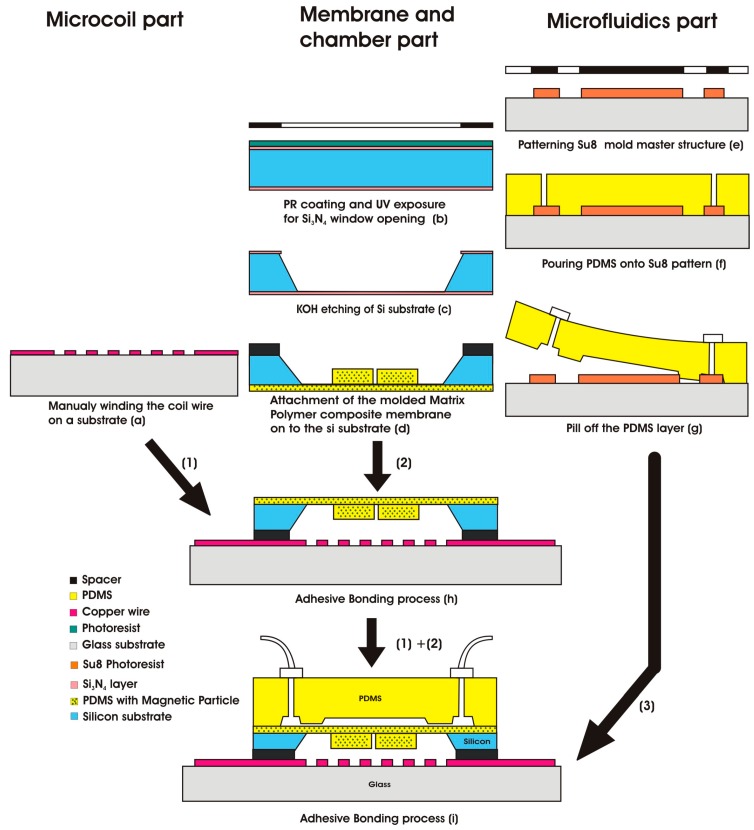
The fabrication process of the EM pump.

The EM pump was fabricated using a standard MEMS process. The process can be divided into three parts: the electromagnetic component, the magneto-mechanic component, and the microfluidic component. Each component of the system was fabricated separately. The step-by-step fabrication process is shown in Figure 6.

The process started with the fabrication of an electromagnetic field generator at the bottom of the actuator (Step 1). A planar spiral coil wire was manually wounded and attached to the glass substrate. The coil wire was used to generate a magnetic field that interacts with the permanent magnet. The diameter of the wire core was 160 µm, and the cladding around the core was about 16 µm. The spacing between the composite membrane and coil was about 0.3 mm. A copper coil was then looped in the planar and glued to the top of the glass substrate.

The fabrication of the actuator membrane started with spin coating the synthesized magnetic polymer composite on to a predefined SU8 mold matrix structure on a glass wafer. The coated polymer composite material was then hard-cured in an oven for 30 min at 60 °C. Finally, the cured polymer composite layers were peeled off and transferred to the top silicon wafer, which was hollow and with dimensions of 6 × 6 mm^2^, to form the actuator membrane (Step 2). To complete the actuator system, the glass wafer with an electromagnetic coil was bonded with the PDMS-based actuator membrane and chamber (Steps 1 and 2).

The microfluidic component was fabricated in a similar way. The process started with pouring the pure PDMS onto a predefined SU8 mold structure. The mold structure was hard-cured and transferred to the actuator membrane surface to form a complete pump system (Step 3).

**Figure 7:**
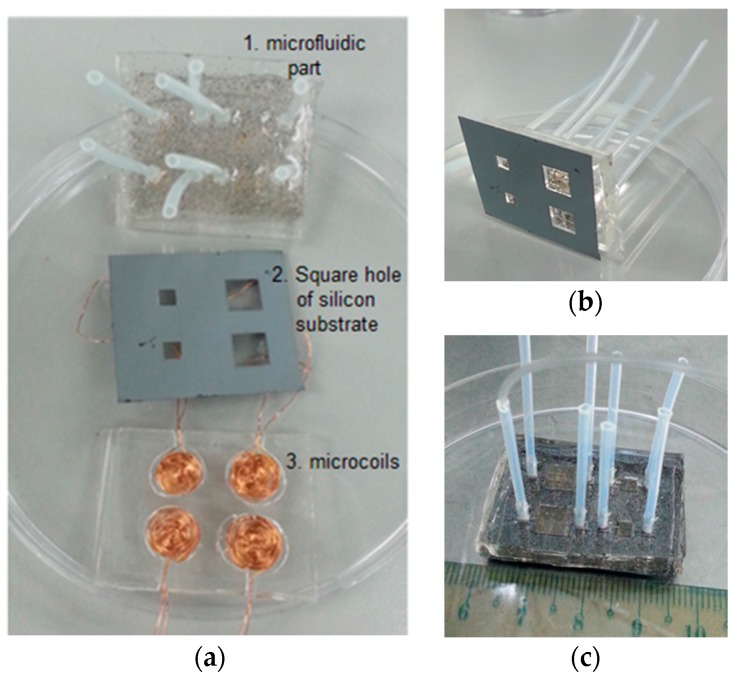
(**a**) The components of the fabricated EM micropump before integration; (**b**) integration of the microfluidic and magnetomechanic components only; and (**c**) the micropump system after integration.

Figure 7a shows the prototype of the developed micropump components before system integration. In Figure 7b, we can see that the magnetic membrane can be properly transferred to a silicon substrate and is ready for the actuation test. Figure 7c shows the complete pump structure after the actuator was bonded with the microfluidic component. The leaking test was conducted in order to ensure that there were no leaks in the chamber or the channel.

**Figure 8:**
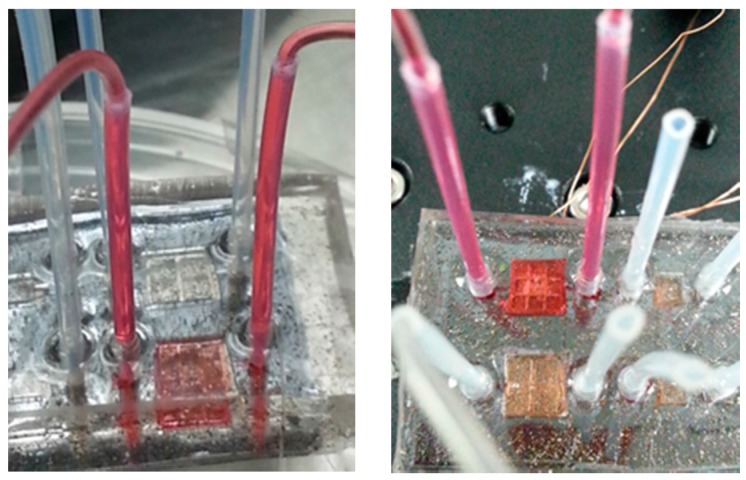
The photographs of the EM micropump under the leaking test.

Figure 8 shows a picture of the tested micropump filled with deionized (DI) water colored red. Red DI water was used to make the liquid flow inside the micropump visible for monitoring purposes. It can be seen in the picture that everything works well. No leaking was spotted.

## 4. Fluid Flow Test of EM Micropump

**Figure 9:**
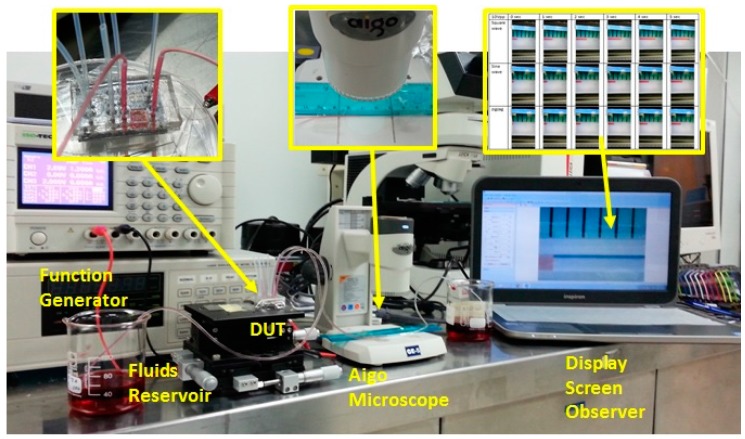
The photograph of the measurement system for the EM micropump test.

The functionality of the micropump was verified using a fluid flow test through the microfluidic channel. The coil was fed with a continuous AC voltage supply by a function generator with controllable frequency. For the measurement of the micropump flow rate, the liquid flow at the outlet tube was monitored using the Aigo digital microscope (Aigo Digital Technology Co., Ltd., Beijing, China). The experimental set up shown in Figure 9 consists of fluid sample storage, fluid tubing connecting the sample storage and the pump, the power supply and control, the Aigo microscope, an observer screen, and the fluid storage at the open end of the fluid tubing on the other side of the pump system.

The functionality test of the fabricated EM micropump was performed by analyzing the flow rate through observation of the liquid flow inside the fluidic tubing for 30 min. The fluid flow was then calculated as the distance of fluid traveling through the fluid tubing with a diameter of 0.37 mm in a certain observation time.

**Figure 10:**
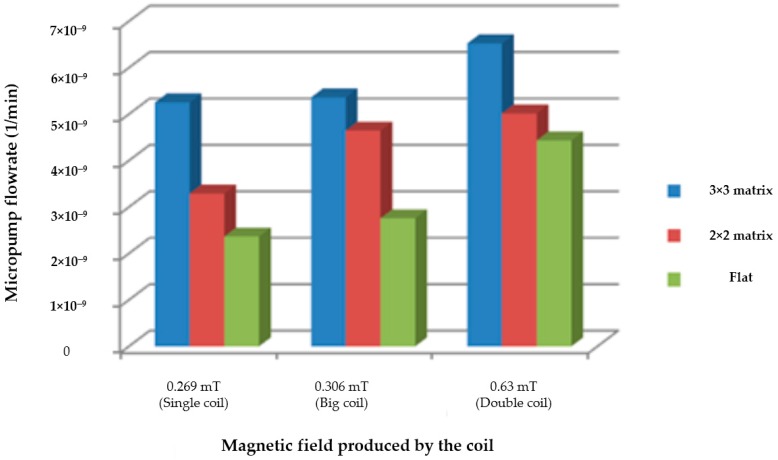
Fluid flow rate versus coil types.

The measurement of the flow rate was taken with the presence of three types of micro-coil, namely, a single planar coil, a larger copper planar coil, and a double planar coil that generates a different magnetic field. The EM micropump with a flat composite membrane and an embossed matrix composite membrane of 3 × 3 and 2 × 2 were tested. An AC supply current from the function generator was fed to the micro-coil. The function generator was set with a 10 Vpp square wave, 0.235 mA, and a 1 Hz operating frequency. The flow rate of each micropump is plotted in the bar graph shown in Figure 10.

It is clearly seen from Figure 10 that the micropump with a membrane matrix of 3 × 3 (the blue bar) had the highest flow rate, 6.523 nL/min. The flat composite membrane (the green bar) had the lowest liquid flow rate for all coils. Moreover, the micropump with the double planar coil, compared to the micropumps with a single planar and a larger planar coil, had a higher flow rate. This is because the magnetic field produced by the double coil (0.63 mT) is the highest among all others. As stated in a previous report [26], the magnetic field generated by the planar electromagnetic coil will directly interact with the permanent magnet and hence produce a magnetic force that pushes and pulls the membrane, allowing for an improved pumping route. These results reflect that a lower membrane deflection will produce a low liquid flow rate.

It can be seen then that the fabricated device was able to deliver fluid at an extremely slow flow rate, below the range reported previously [6]. It was also shown that the fluid flow characteristic was proportionally related to membrane deformation. Therefore, the injection flow rate could be controlled by adjusting the frequency, the signal type, and the height of the input power supplied to the electromagnetic coil. We showed here that magnetic fields generated by microcoils and a smaller matrix membrane design can be used to significantly control the fluid flow rate of the micropump. Such a controlled flow rate is believed to have very high potential in enhancing fluid injection system capability for continuous drug delivery.

## 5. Conclusions

In this work, an EM micropump with a matrix composite membrane was successfully designed, fabricated, and tested. The composite is a mixture of PDMS and 6% NdFeB particles and designed to be an embossed matrix membrane for the micropump. The design eradicates the need for bulk magnets, allowing for a more compact and simple design. Embossed matrix membranes of 1 × 1, 2 × 2, and 3 × 3 were built, and the 3 × 3 matrix membrane produced the highest membrane deflection value, 12.87 µm. As a micropump, the 3 × 3 matrix membrane attained the highest flow rate of 6.523 nL/min when the micropump was driven by a double planar coil. The designed EM micropump has been successfully used with nano-dose sample injection and thus is highly desirable for applications that require very high injection resolutions for high accuracy dosages, especially in lab-on-chip and drug delivery systems.

## Figures and Tables

**Table 1:** The dimension of the designed polymer composite membrane.

Membrane Type	Dimension	Membrane Type	Dimension
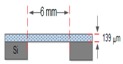	6% NdFeB Shape: Square flat. Area/cell: 6 × 6 mm^2^ Thick: 139 µm	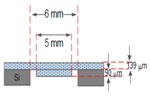	6% NdFeB Shape : Square (1 × 1) Area/cell: 5 × 5 mm^2^ Thick:~ 90 µm
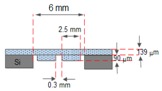	6% NdFeB Shape: Square (2 × 2). Area/cell: 2.5 × 2.5 mm^2^ Thick: ~90 µm	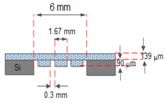	6% NdFeB Shape: Square (3 × 3) Area/cell: 1.66 × 1.66 mm^2^
